# Silver decorated lithium niobat nanostructure by UV activation method for silver–lithium niobate/silicon heterojunction device

**DOI:** 10.1038/s41598-023-38363-8

**Published:** 2023-07-17

**Authors:** Evan T. Salim, Wafaa K. Khalef, Makram A. Fakhri, Rawan B. Fadhil, Ahmad S. Azzahrani, Raed Khalid Ibrahim, Raid A. Ismail

**Affiliations:** 1 Applied Science Department, University of Technology-Iraq, Baghdad, Iraq; 2 Laser and Optoelectronic Engineering Department, University of Technology-Iraq, Baghdad, Iraq; 3 Electrical Engineering Department, Northern Border University, Arar, KSA Saudi Arabia; 4 AlFarahidi University, Baghdad, Iraq

**Keywords:** Nanoscience and technology, Nanoscale materials

## Abstract

Lithium niobite (LiNbO_3_) nanostructure were successfully synthesized by chemical bath deposition method (CBD) and then decorated with silver nitrate (AgNO_3_) through UV activation method at different immersion durations (5, 15, 25, 35, and 45 s). The silver nanoparticles (AgNPs) effects on the optical and structural properties were studied and analyzed using various scientific devices and technique. X-ray diffraction (XRD) results showed that all the samples have a hexagonal structure with a maximum diffraction peak at the (012), and the existence of silver atoms could be recognized at 2θ = 38.2° which corresponds to the (111) diffraction plane. The optical absorption of nanocomposites depicted the presence of plasma peak related to silver (Ag) at 350 nm. The estimated energy gap from the optical absorption revealed a reduction in the Eg value from (3.97 eV) to (3.59 eV) with the presence of Ag atom. The Photolumincence (PL) peaks were observed at around 355 nm for pure LiNbO_3_/Si and 358, 360, 363, 371, 476 nm for different immersion durations respectively, in the visible region of the electromagnetic spectrum. The scanning electron microscopy (SEM) study illustrated that with increasing the immersion time, especially at 45 s, a change in the particle morphology was observed (LiNbO_3_ NRs structure). Atomic force microscopy (AFM) displayed that the surface roughness decreases from 80.71 nm for pure sample to 23.02 nm for the decorated sample as the immersion time is increased. FT-IR manifested a noticeable increase in the intensity of the peaks of samples decorated with AgNPs. Raman spectroscopy elucidated that the peaks shifted to higher intensity due to the plasmonic effect of Ag nanoparticles. Ag–LiNbO_3_/Si heterojunction nano-devices were fabricated successfully and enhanced the optoelectronic properties in comparison with the pure LiNbO_3_/Si heterojunction device.

## Introduction

Lithium niobate (LiNbO_3_) is an inorganic material that has been widely studied in the past decades^1–3^. Due to its excellent acoustic-optical, piezoelectric, pyroelectrical electro-optics, and photo-refractive properties, it is a significant optical material that is widely used in the Photonics industry; due to its significant second-order nonlinearities, it is one of the most effective materials for electro-optic application^4–8^. Owing to its performance, LiNbO_3_ is a promising candidate for building optoelectronic tweezers^9–12^, optical waveguides, optical modulators, Pockels cells^13^, and optical parametric oscillators^14^. The successful integration of LiNbO_3_ into optoelectronic components and devices includes fiber-based communication, wireless communication, and micro-electromechanical systems (MEMS)^15,16^. Hence, researchers have paid a particular attention to the synthesis of LiNbO_3_ through different techniques, such as combustion methods^17^, sol–gel, hydrothermal^18–20^, wet chemical methods^21^, molten salt methods^22,23^, surfactant assisted solution-phase methods^24^, pulsed laser deposition (PLD) and pulse laser ablation (PLA)^25–29^, as well as the chemical hydrothermal technique and CBD^30,31^. The LN films on Si semiconductors are particularly desirable for the development and production of integrated ferroelectric, photonic, and sensing devices due to their combination of exceptional features. Recently, fundamental research and technical applications, such as electrical devices, gas sensors, and catalysts have acknowledged the great relevance of the notion of surface plasmon-based photonics, or "plasmonics," in metal on the metal oxide semiconductor systems^15,32–35^. Noble metal nanoparticles (NPs), such as Au^36,37^, Ag^38,39^, Cu^40^, Pt^41^, Pd^42^, and Ru^43^, have been the subject of intense research recently because, in addition to their superior chemical, optical, mechanical, and electrical properties, they also exhibit good catalytic and biological activities. Among them, AgNPs, a prospective noble metal, offer attractive qualities, including cheap cost, excellent conductivity, and chemical stability, as well as they have demonstrated the potential uses in energy and optoelectronics^44–46^. AgNPs are also frequently employed to increase the UV–visible light absorption due to their distinct electron confinement, which results in a localized surface Plasmon resonance (LSPR). When the wavelength of the input light beams surpasses their size, the photo-induced conductive electrons on the surface of metal nanoparticles (NPs) oscillate together to generate this effect^47,48^. A Schottky barrier may be effectively formed using a suitable semiconductor to collect these electrons.

Previous published studies were found about decorating different oxide semiconductors with various materials. Among these studies, L.W. Zainuddin et al. Investigated the electronic and optical properties of Ag and Au doped LiNbO_3_. The enhancement in the optical absorption of Ag and Au doped LiNbO_3_ made it a promising material for photovoltaic and photocatalyst application^49^. Marwa S. Alwazny et al. prepared hybrid and novel Gold core Lithium niobate shell (Au@LiNbO_3_, Au@LN) nanoparticles by two steps of laser ablation in liquid. Structural and optical properties exhibited an enhancement of Au@LiNbO_3_. The results of the photodetectors were correlated with the optical, structural and electrical properties of Au@LiNbO_3_ nanostructure^50^. Hao et al. examined the effects of the buffer zinc oxide (ZnO) film on the capabilities of the pulsed laser-deposited LiNbO_3_/n-Si heterojunction photodetector^51^. Evan T. Salim et al. incorporated silver NPs into Nb_2_O_5_ nano matrix structure by photo activation mechanism. The enhanced Nb_2_O_5_ thin films by the plamonic effect of silver noble metal NPs were employed in fabricating a p–n heterojunction photodetector^52^. Li et al. showed how to create an iron-doped (Fe-doped) LiNbO_3_/n-Si heterojunction for use in integrated optics and electro-Photonics^53^. However, no work has been reported on the Ag-decorated lithium niobate nanostructure Ag–LiNbO_3_/Si heterojunction device. In this paper, a very simple, low cost, chemical bath deposition method has been used for the synthesis of LiNbO_3_ nanostructure, and the produced LiNbO_3_ nanostructure with the UV-activation plasmonic effect of silver (Ag) nanoparticles by soaking the LiNbO_3_ in the AgNO_3_ solution has been enhanced to show the effect of adding silver in improving the electro-optic properties of LiNbO_3_. A chemical bath deposition method and the silver nanoparticles decoration of LiNbO_3_ nanostructure in this study have been performed for the first time for the best of researcher's knowledge. A pn-photodetector has been fabricated based on the obtained results.

## Experimental

**Figure 1:**
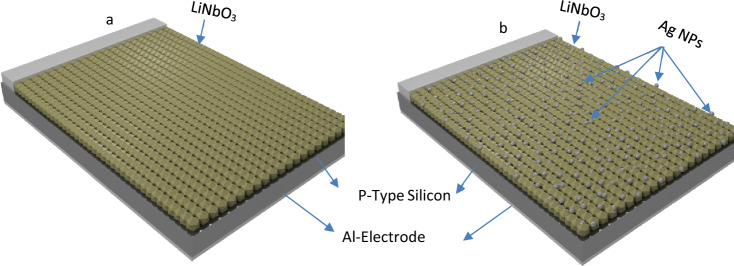
The schematic diagram of both heterojunction devices (**a**) (LiNbO_3_/Si) and (**b**) (Ag decoration LiNbO_3_/Si).

Without further purification, citric acid (CA) and ultra-pure (99.99%) niobium pentoxide were utilized. A solution was created by combining CA and ethylene glycol (EG) in a glass baker for two hours. Once it reached 90 °C, the stirring of Li_2_CO_3_ and Nb_2_O_5_ was kept for 6 h. The following materials were utilized: Li_2_CO_3_ = 3.7 g, Nb_2_O_5_ = 13.30 g, CA = 10.5 g and EG = 20 g. The molar ratio between Nb_2_O_5_ and Li_2_CO_3_ according to the procedures utilized in a previously published paper was 1:1^54–57^. In one beaker, the ingredients were combined. The samples were made by applying a film to the quartz substrate using the CBD technique for 15 min after 12 h. The solution beaker was filled with the quartz substrate standing up. To remove the organics, all of the produced films were finally annealed at 500 °C for 2 h in a static air and oxygen environment. The photo-reduction process included a sequential immersion in AgNO_3_ solution followed by UV illumination^58,59^. By using the UV activation approach, which initially submerged the LiNbO_3_ thin films into a 1 M solution of AgNO_3_ for varying lengths of time (5, 15, 25, 35, or 45 s), Ag was added to the thin films. The film was illuminated with UV radiation from a higher value of Halogen lamp for 15 min to add the photo-reduction of silver ions to the metal. X-ray diffraction (XRD) (Schimadzu 6000)-type instrument was used to investigate the structural properties of LN. Scanning electron microscopy (SEM) (InspectTM F50) and atomic force microscopy (Angstrom advanced lnc.) were employed to examine the morphology of LN's surface. The double-beam ultraviolet (UV)-visible spectrophotometer (Schimadzu 1800)-type equipment and Fourier transforms infrared spectroscopy (Bruker 7613)-type instrument were used to examine the optical characteristics. The exactness impedance analyzer supplied from (Agilent, 4294A, USA) was used to study the C–V properties. Figure 1 shows the schematic diagram of both heterojunction devices (LiNbO_3_/Si) and (Ag decoration LiNbO_3_/Si).

## Results and discussion

### structural properties

**Figure 2:**
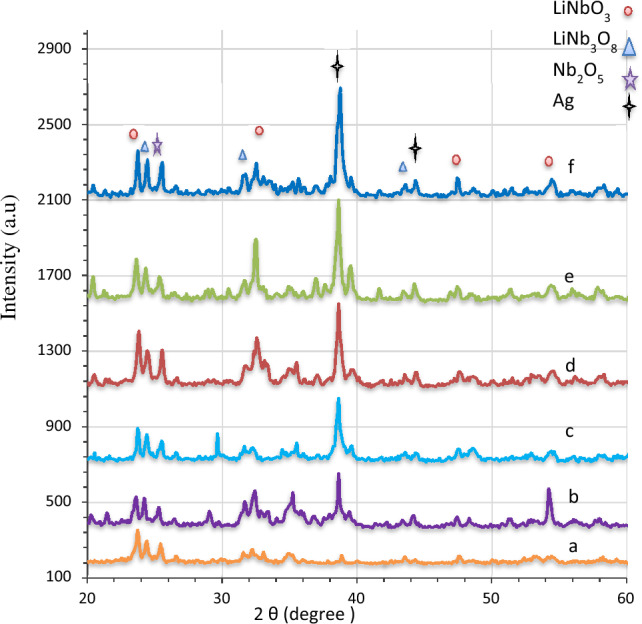
XRD diffraction patterns of LiNbO_3_ nanostructure (**a**) pure, decorate nanostructure at (**b**) 5 s, (**c**) 15 s, (**d**) 25 s, (**e**) 35, (**f**) 45 s.

The X-ray diffraction results for pure and Ag-decorated LiNbO_3_ nanostructure were obtained using various immersion times. As shown in Fig. 2, the LN peaks at 2θ = 23.68°, 32.64°, 34.62°, 47.54° and 54.31° correspond to the (012), (104), (110), (024) and (116) planes, respectively^60–65^. Table 1 lists the structural properties of pure and Ag-decorated LN nanophotonics that must be measured. Scherrer’s formula was used to compute the crystallite size (D)^66–68^.

1 $$D=\frac{K\lambda }{\beta \, \mathrm{cos} \, \theta }$$

where, the constant k is assumed to be 0.94, λ is the utilized X-ray wavelength that is assumed to be 1.54 Å, and θ is the Full width at a half maximum of the X-ray diffraction pattern equal to Bragg's angle. In order to calculate the strain (ε) and dislocation density (δ) of LN nanophotonics, the following relationships were used^69–75^.

**Table 1:** Ag-decorated LiNbO_3_ nanophotonic parameters at different decorated time.

Chemical interaction time (min)	Orientation (hkl)	Peaks (2θ)	Grain size (nm)	Dislocation Density (δ) (10^9^) (lines/m^2^)	Strain (10^−3^)	dhkl	Lattice constants a, c
Pure	12	23.75	102.67	9.48	0.68	3.76	a = 5.1566
							c = 13.85
	104	32.6	96.52	10.73	0.37	2.74	a = 5.1566
							c = 13.85
	110	34.55	93.08	12.88	0.31	2.58	a = 5.1566
							c = 13.85
	24	47.4	91.02	17.3	0.28	1.88	a = 5.1566
							c = 13.85
	116	53.23	85.15	21.53	0.26	1.72	a = 5.1566
							c = 13.85
5 s	12	23.68	95.41	11.45	0.64	3.74	a = 5.1566
							c = 13.85
	104	32.6	93.12	14.12	0.54	2.73	a = 5.1566
							c = 13.85
	110	34.64	89.91	15.98	0.33	2.57	a = 5.1566
							c = 13.85
	24	47.54	88.19	17.63	0.27	1.87	a = 5.1566
							c = 13.85
	116	54.31	81.62	19.23	0.25	1.71	a = 5.1566
							c = 13.85
15 s	12	23.74	93.8	12.98	0.59	3.75	a = 5.1566
							c = 13.85
	104	32.6	91.64	15.24	0.5	2.71	a = 5.1566
							c = 13.85
	110	34.65	88.2	18.11	0.3	2.57	a = 5.1566
							c = 13.85
	24	47.54	87.32	20.67	0.26	1.86	a = 5.1566
							c = 13.85
	116	54.31	79.93	21.87	0.23	1.68	a = 5.1566
							c = 13.85
25 s	12	23.75	92.24	14.45	0.55	3.74	a = 5.1566
							c = 13.85
	104	32.61	89.24	16.27	0.46	2.7	a = 5.1566
							c = 13.85
	110	34.65	84.85	19.68	0.29	2.54	a = 5.1566
							c = 13.85
	24	47.55	83.98	22.49	0.25	1.91	a = 5.1566
							c = 13.85
	116	54.32	77.87	23.72	0.23	1.68	a = 5.1566
							c = 13.85
35 s	12	23.76	88.12	15.14	0.42	3.74	a = 5.1566
							c = 13.85
	104	32.61	85.96	16.89	0.33	2.73	a = 5.1566
							c = 13.85
	110	34.65	81.85	20.19	0.27	2.57	a = 5.1566
							c = 13.85
	24	47.57	78.99	23.11	0.24	1.87	a = 5.1566
							c = 13.85
	116	54.36	76.61	24.29	0.22	1.71	a = 5.1566
							c = 13.85
45 s	12	23.7	86.32	16.91	0.35	3.75	a = 5.1566
							c = 13.85
	104	32.62	83.68	18.25	0.31	2.7	a = 5.1566
							c = 13.85
	110	34.55	80.97	22.68	0.25	2.55	a = 5.1566
							c = 13.85
	24	47.75	76.56	23.17	0.23	1.88	a = 5.1566
							c = 13.85
	116	53.24	73.85	25.37	0.21	1.67	a = 5.1566
							c = 13.85

2 $$\delta =\frac{1}{{D}^{2}}$$

3 $$\varepsilon =\frac{\beta } {4 \,{\tan} \, \theta }.$$

Bragg’s formula was used to compute the interplanar distance (d) for the all sets of LN nanophotonics^76–79^.

4 $$d=\frac{n}{2 \, \mathrm{ sin \, \theta }},$$

where, n is a positive integer number, and d is the value provided in Table 1. Two phases of LN with a polycrystalline structure may be identified in nanophotonics, i.e. LN and LiNb_3_O_8_ phases. The LN phase is preferable. However, the achieved phase is oriented at (012). From the XRD results, a small amount of secondary lithium-deficient phase (LiNb_3_O_8_) is clearly observed in the all samples. This phase is produced through the oxygen–LiNbO_3_ interfacial interaction. The XRD peaks of LiNb_3_O_8_ are at 2θ = 24.45, 31.10, 35.20, 43.47 corresponding to the (400), (202), (− 601), (203) and planes, respectively. These results agree with the Joint Committee on Powder Diffraction Standards (JCPDS) card no. 01-074-2239. Notably, the different peaks (Nb_2_O_5_) occur at 2θ = 25.55, which corresponds to the (− 212) plane. This result concurs with JCPDS card no. 01-074-2239).

The diffraction peaks of nano silver at 2θ = 38.2, which corresponds to the (111) diffraction plane, as portrayed in Fig. 2, might be used to identify the presence of silver atoms on the LiNbO_3_ nanostructure. In spite of the presence of the main phases related to LiNbO_3_ nanostructure, their intensities demonstrated a consistent lowering with an increase in the silver incorporation. A notable diffraction peak connected to the (200) diffraction plane could be seen. The Ag nanoparticles at 2θ = 44.3 diffraction angle account for this peak^80^.

According to a previous study, a rise in the peak intensities assures that the Ag nanoparticles are incorporated into the base material's lattice^52^. Table 1 indicates that the experimental values and measured lattice constants are similar^81^.

### Optical properties

**Figure 3:**
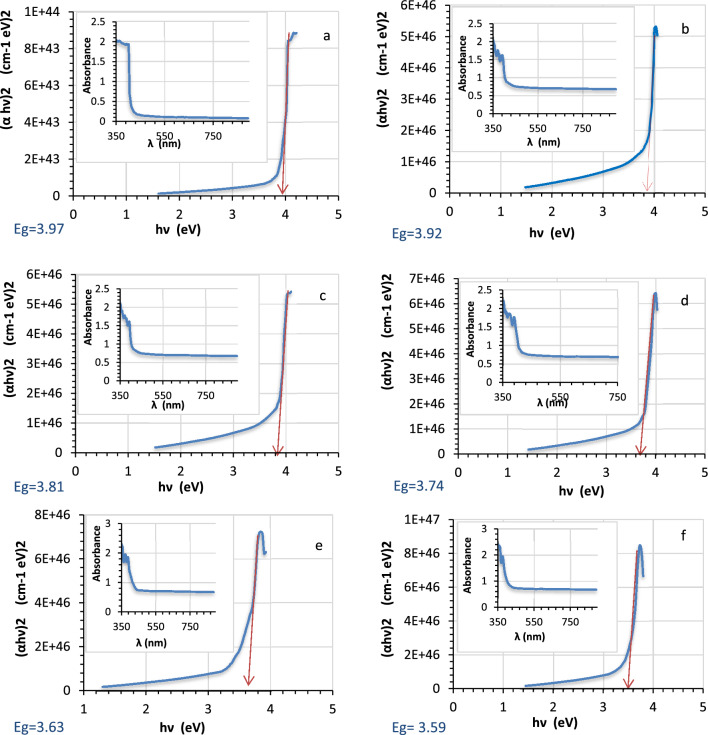
Energy gap and absorption of (**a**) pure LiNbO_3_, Ag-decorated LiNbO_3_ (**b**) 5 s, (**c**) 15 s, (**d**) 25 s, (**e**) 35 s, (**f**) 45 s.

The optical properties results for pure and Ag decorated LiNbO_3_ nanostructure using various immersion times (5, 15, 25, 35, 45 s) are illustrated in Fig. 3. The absorption peaks ranging from 350 to 400 nm at the region of UV are one of the unique characteristics of the LiNbO_3_ nanostructure, and this result confirms the results of X-ray diffraction (XRD) shown previously. The optical absorbance increases by 50% as the number of the nanoparticles of silver are growing by adjusting the immersion period, as depicted in Fig. 3. The increases in the silver (Ag) decorating time are seen at 400 nm and might be attributed to the plasmonic effect. Also, this was ascribed to the charge transfer transition between the electrons of Ag and LiNbO_3_ which could reduce the band gap energy between the conduction band and the valence band of LiNbO_3_, and the Ag nanoparticles on LiNbO_3_ can trap the electrons resulting in prevention of the electron hole recombination^82^.

**Figure 4:**
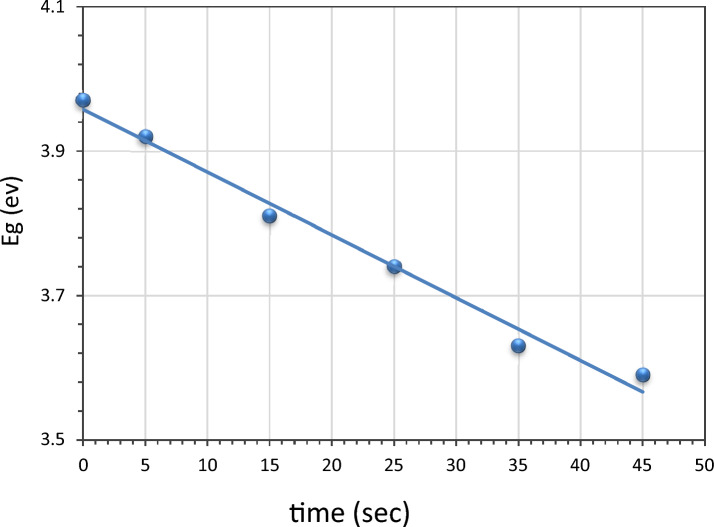
The energy gap changes with varying immersion times.

The calculated energy gap is revealed against the immersion time in Fig. 4. One observes that the energy gap of LiNbO_3_ was 3.97 eV. After Ag decoration LiNbO_3_, the band gap energy was reduced to 3.59 eV. It is clear that the effects of the quantum confinement in the films cause the optical energy gap to narrow with increasing the Ag (silver) concentration. With added silver, the band gap energy drops. Since the LiNbO_3_'s conduction band edge may experience a band bending due to the close contact between the Ag nanoparticles and the host material, a reduction in the optical band gap energy of LiNbO_3_ is anticipated to occur^83^.

### PL measurements results

**Figure 5:**
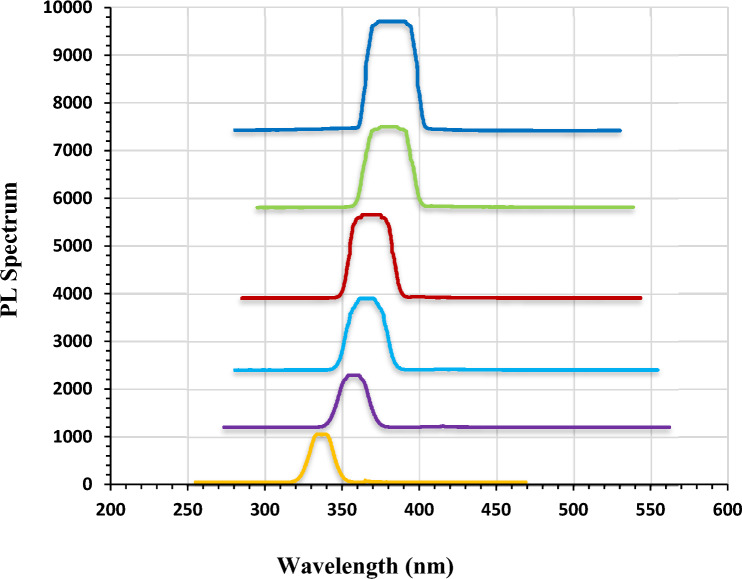
PL spectra of (**a**) pure and decorated films at different immersion time (**b**) 5, (**c**) 15, (**d**) 25, (**e**) 35, (**f**) 45 s.

The photoluminescence (PL) spectra of the pure and Ag-decorated LiNbO_3_ nanostructure grown on the quartz substrate at different immersion times are displayed in Fig. 5. The PL peaks are observed at around 335 (for pure LiNbO_3_), 358 (for 5 s Ag-decorated LiNbO_3_), 360 (for 15 s Ag-decorated LiNbO_3_), 363 (for 25 s Ag-decorated LiNbO_3_), 371(for 35 s Ag-decorated LiNbO_3_) and 476 (for 45 s Ag-decorated LiNbO_3_) in the visible region of the electromagnetic spectrum. These are consistent with the X-ray diffraction (XRD) results and the optical properties which are shown previously. The emission intensity is significantly weakened in pure LiNbO_3_ compared to the decorated films, and the band shifts slightly towards the higher wavelength region. This is attributed to the band to band transition of electrons from the conduction band to the valence band. This indicates that the recombination rate of photo-generated carriers increased when doping with Ag. These results agree with^84^.

### Morphological studies

**Figure 6:**
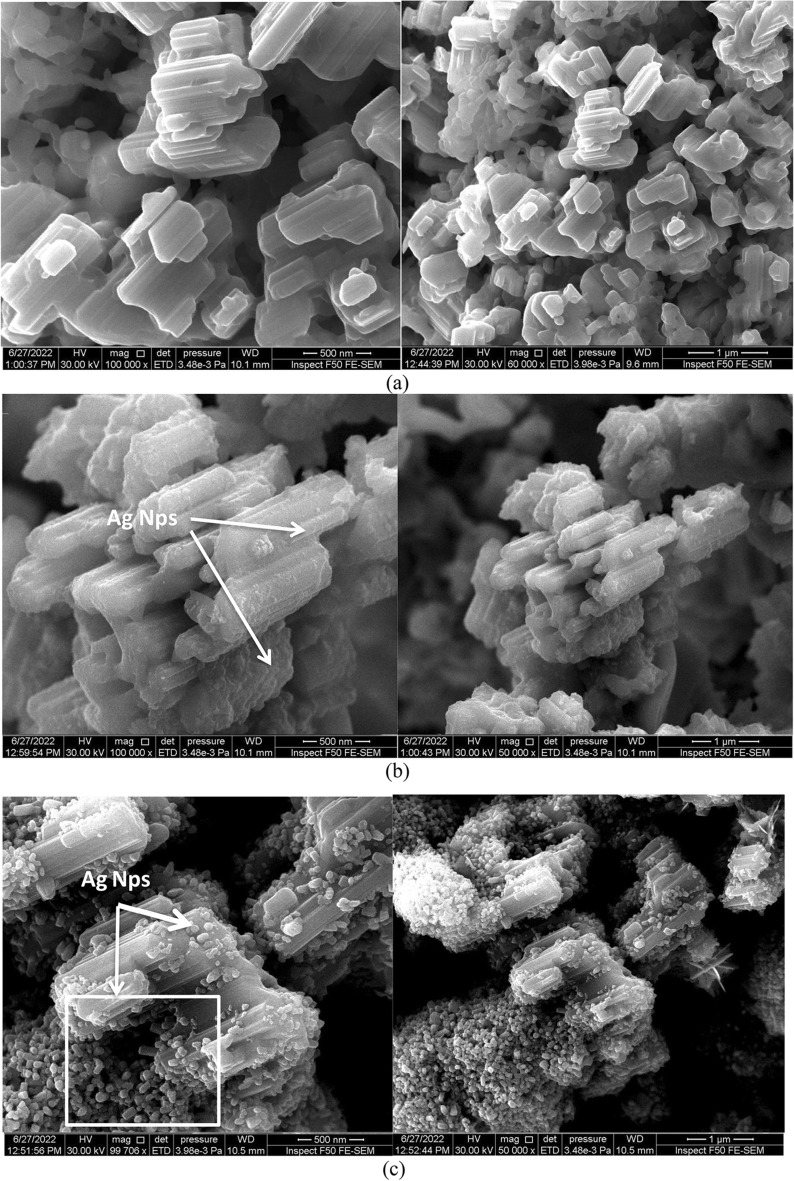

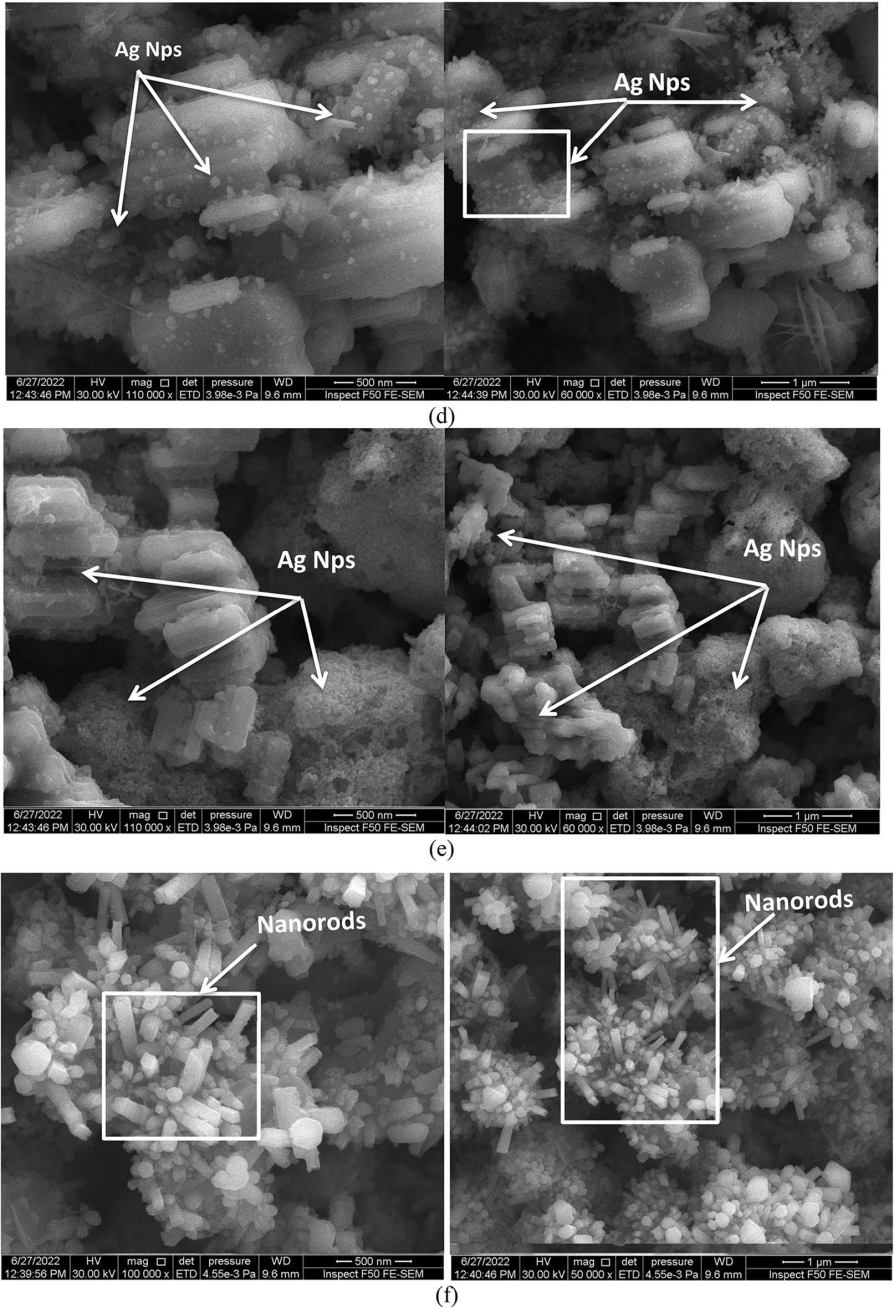
FE-SEM image of (**a**) pure LiNbO_3_ Ag-decorated LiNbO_3_ at (**b**) 5 s, (**c**) 15, (**d**) 25 s, (**e**) 35 s, and (**f**) 45 s.

SEM was used to determine the surface morphology of the LiNbO_3_ nanostructure surface at various immersion times for the pure and Ag-decorated LiNbO_3_ nanostructures, as manifested in Fig. 6a–e. The pure LiNbO3 nanostructure in Fig. 6a views the smooth and homogenous structure. The crystalline size was calculated from the FESEM image and was about 100–110 nm for pure LiNbO_3_ and around 95, 90, 85, 80, and 75 nm for Ag decoration LiNbO_3_ at 5, 15, 25, 35, and 45 s respectively. The number of Ag nanoparticles rises with the doping time of the final immersion periods, as seen in Fig. 6b–f. The measured structural characteristics concur with this. Their sizes and shapes were consequently changed. As nanoparticles possess a larger surface area in comparison with their volume, their surface energies are high and these particles agglomerate to reduce the excessive surface energies and reach a thermodynamic stability condition. The agglomeration process is majorly occurring as a result of Van der Waals attraction^52,85^. It was found that with increasing the immersion time, especially at 45 s, a change in the particle morphology and size occurs, as evinced in Fig. 6. Small spherical grains that are part of the Ag nanoparticles are submerged for shorter periods of time, whereas the creation of the Ag nanorods may be detected after 45 s. This may be due to the sliver spot can be localized at the top end of growing nanorods called the tip growth process. This can follow the vapor–liquid–solid (VLS) mechanism.

**Figure 7:**
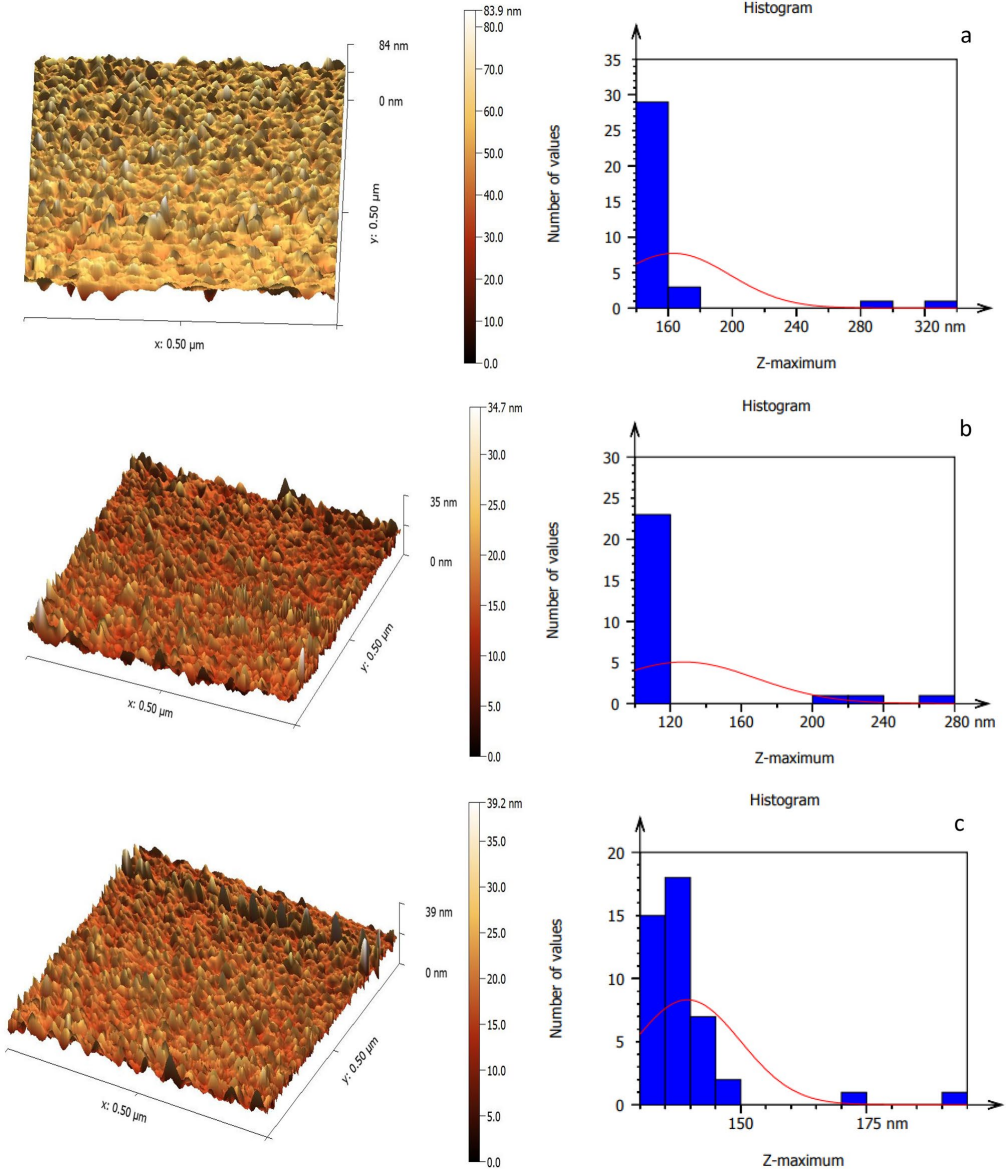

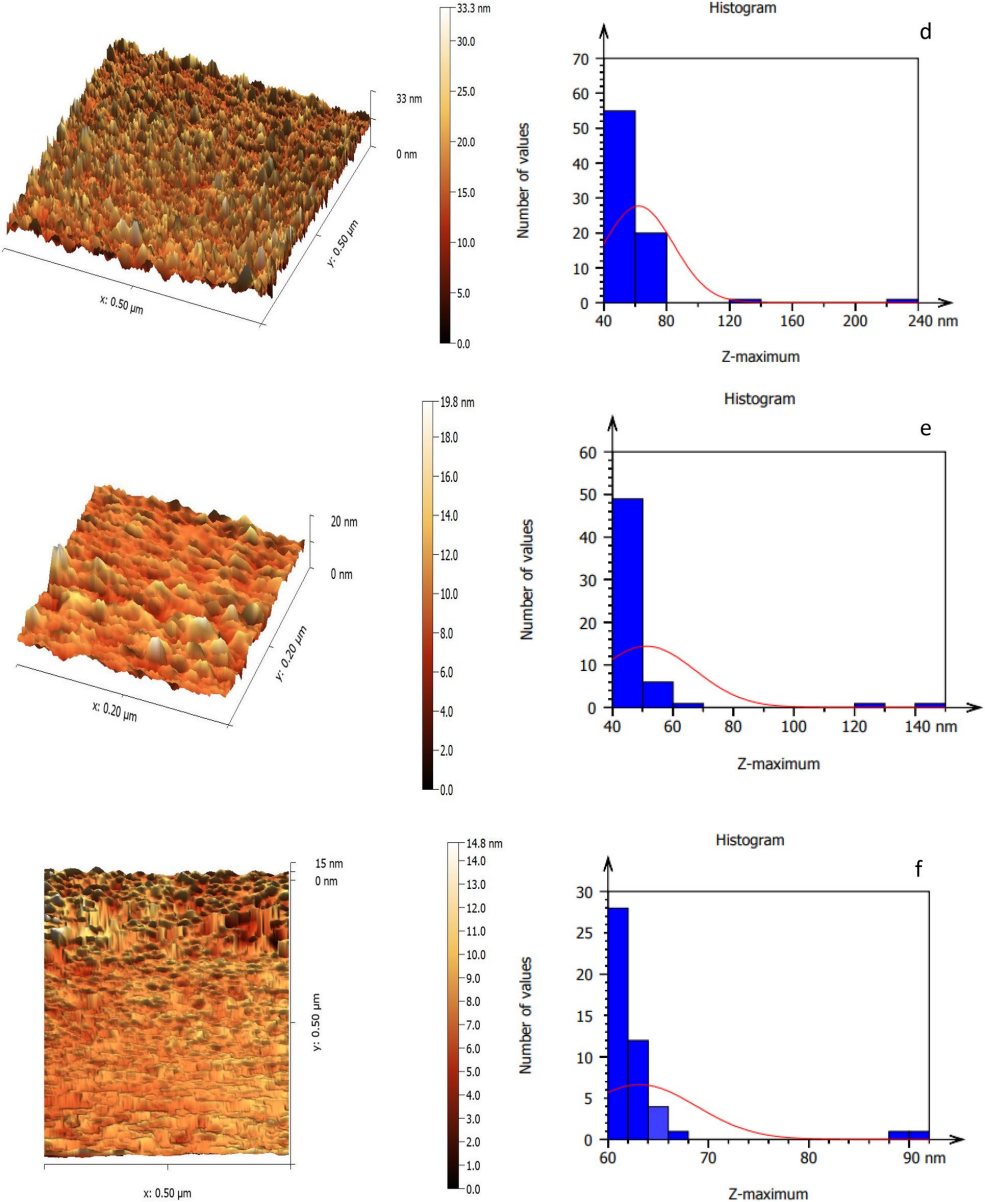
AFM images of (**a**) pure LN, Ag-decorated LN at (**b**) 5 s, (**c**) 15 s, (**d**) 25 s, (**e**) 35 s and (**f**) 45 s.

Figure 7 portrays the AFM pictures of the LiNbO_3_ nanophotonics, which have a surface with a homogeneous density and demonstrate a reduction in grain size with increasing immersion duration^86^. The LiNbO_3_ nanophotonics' surface topography, as elucidated in the AFM micrographs, demonstrates that the grains are evenly dispersed across the scanning region. LiNbO_3_ topographical images were not significantly changed when decorated by silver (Ag) nanostructures that was due to the sparsed distribution of the small spherical silver nanoparticles within LiNbO_3_ nanostructure outermost layers. However, the histograms revealed more obvious results. On the other hand, it was noted that the surface roughness decreases from 80.71 to 23.02 nm as the immersion time is increased. The root mean square also decreases from 91.61 to 28.14 nm. Increasing the Ag decoration levels leads to a decreasing in the root mean square to reach a minimum value of 28.14 nm at 45 s. According to this result, the reduction in the surface roughness in the Ag decoration levels may be attributed to the decrease of LiNbO_3_ grain size, which was discussed previously, and this result is in a good agreement with^27^.

### Fourier transform infrared spectroscopy

**Figure 8:**
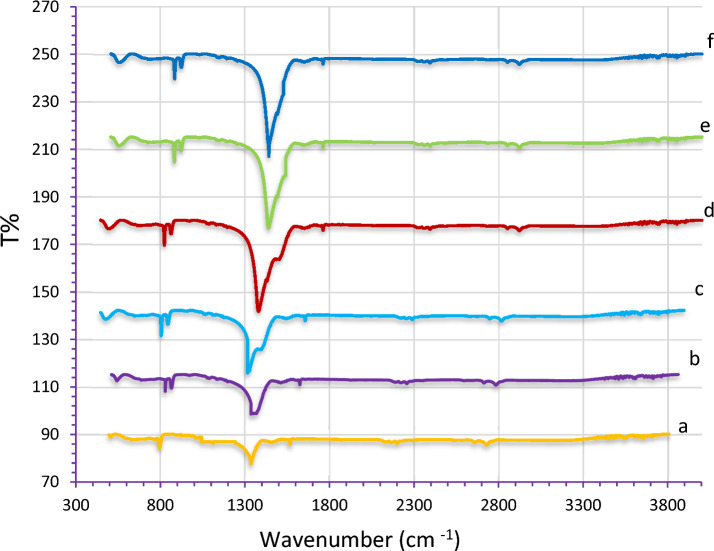
FTIR spectrum of (**a**) pureLiNbO_3_, Ag-decorated LiNbO_3_ at (**b**) 5 s, (**c**) 15 s, (**d**) 25 s, (**e**) 35 s and (**f**) 45 s.

The FTIR spectra of pure LiNbO_3_ and Ag-decorated LiNbO_3_ are shown in Fig. 8. These results showed that LiNbO_3_, grown crystal were homogeneous in composition. The figure shows the infrared spectra of different immersion time in AgNO_3_. It can be observed that the band at 515 cm^−1^ is related to specific vibration of Li–O bonds. Also can be observed in all samples that the band 871 cm^−1^ is assigned to Nb–O–Nb stretching vibration^87^. The absorption peaks at 1470 cm^−1^ are assigned to bending vibration of surface adsorbed water molecules. From the results, we notice that the bonds between the materials are formed. No bands associated with the additional phases of AgO or AgO_2_ are visible in FTIR spectra, which points to the great purity of the produced materials. A noticeable increase in the intensity of the peaks of samples doping with Ag Nps. The change of O–H stretch absorption intensity in the composite sample is attributed to the interactions between silver ions and hydroxyl group of LN and may be attributed to presence of Ag nanoparticles due to the effect of surface plasmon resonance (SPR), this results agree with^88^.

### Raman spectra measured

**Figure 9:**
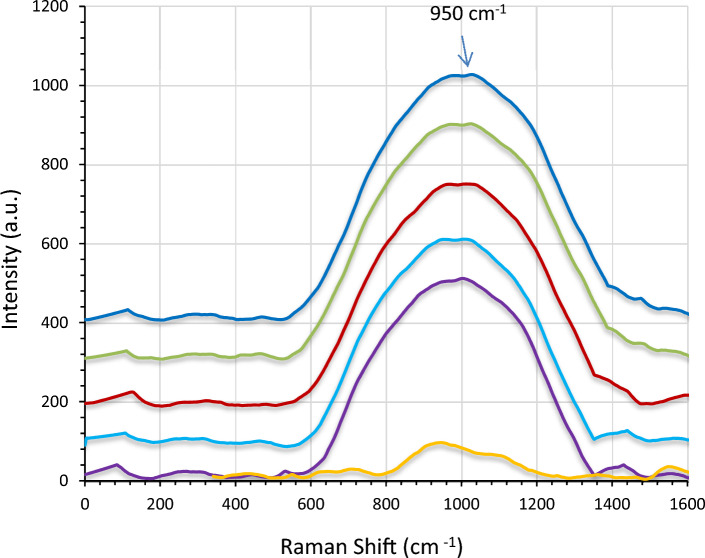
LiNbO_3_ Raman spectra for (**a**) pure and decorated film at (**b**) 5, (**c**) 15, (**d**) 25, (**e**) 35 and (**f**) 45 s.

Figure 9 illustrates the Raman spectra with the wave numbers ranging from 200 to 1000. The peaks for pure LiNbO_3_ were observed at 258, 410, 510, 745 and 830 cm^−1^ and were attributed to the LN phonon mode E transverse optical (TO). After being decorated with AgNPs, it was noticed that the peaks shifted to a higher intensity due to the plasmonic effect of Ag nanoparticles. To explain that, the Raman enhancement occurred as a result of the hot spot's impact. The hot spots stand for the gaps between the metallic nanoparticles, when exposed to a ram of an incident source, exhibit high intense bands due to the enhanced electric field resulting from the plasmonic effect of Ag nanoparticles. This can be clearly noted for the band observed in the wave number region exceeding 1000 cm^−1^ that might be belong to Ag.

### Resistance (R) with temperature (T)

**Figure 10:**
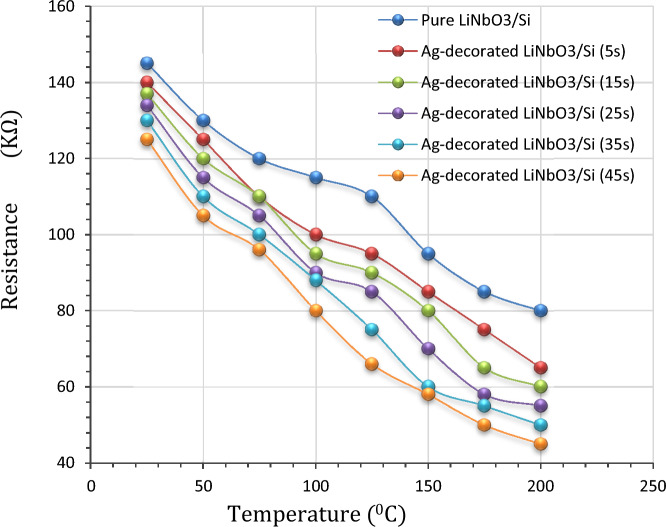
Pure and Ag-decorated LiNbO_3_ resistance as a function of temperature.

Figure 10 views the resistance (R) of pure and Ag-decorated Lithium niobate as a function of temperature which was measured by Kiethly electrometer. Each sample was placed on a plate under which a furnace like structure was equipped. The first reading for all the samples was taken under thermal equilibrium condition (at room temperature) to test the resistance of LiNbO_3_ nanostructure prepared with different films’ thicknesses. The minimum obtained resistance was about 45 kΩ. Two point’s probes were attached to the metalized zones. The range of temperature began from the room temperature up to 200 °C. The relative resistance of all samples decreases continuously with increasing temperature after being decorated with silver nanoparticles. Such behaviour can be attributed to the increasing electrical conductivity with increasing temperature due to the electron transition from the valence band to the conduction band^50^.

### Figure of merit of Ag at LiNbO_3_ nanostructure

**Figure 11:**
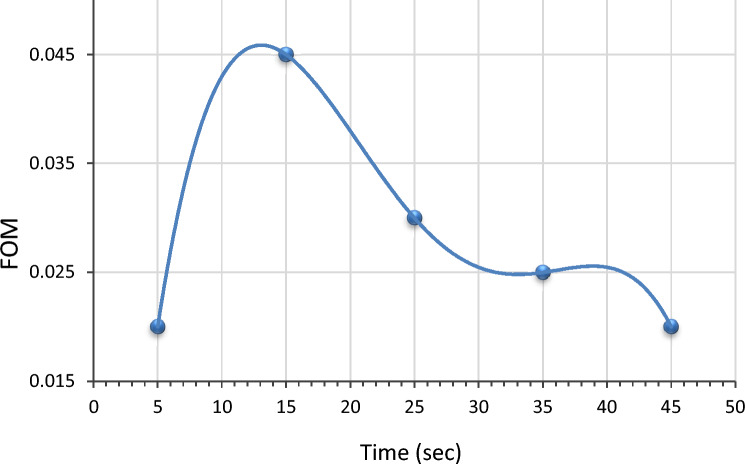
The change of figure of merit with immersion time.

Figure 11 depicts the figure of merit (F.O.M.) of LiNbO_3_ thin films prepared after multi-optimization steps. Different immersion times were used to prepare Ag at the LiNbO_3_ nanostructure sample. The applicability of prepared films in optoelectronic devices could be quantified by the optimal combination of high electrical conductivity and high absorption of visible light, and the figure of merit (ɸ) can be calculated by applying^89,90^:

5 $$\phi = {1}/\alpha \rho ,$$

where, α refers to the absorption coefficient, and ρ is the electrical resistivity. The figure of merit used in judging the quality of the immersion time for the prepared films can be observed in Fig. 11 that shows the changing of the figure of merit with the immersion time, and it was found that the best immersion time is at 15 s.

## Electrical properties of LiNbO_3_/ Si and Ag decorated LiNbO_3_/Si heterojunction device

### Current–voltage (I–V) measurement

**Figure 12:**
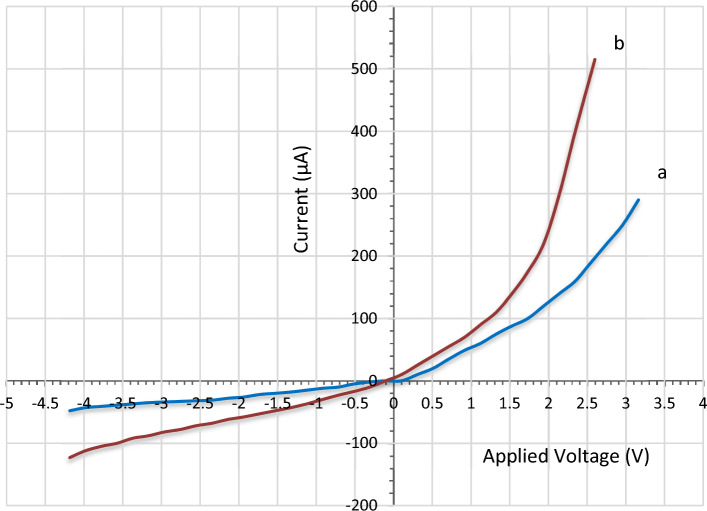
I–V characteristics of (**a**) pure LiNbO_3_/Si and (**b**) Ag-decorated LiNbO_3_/Si heterojunction device.

The influence of adding of silver NPs on the optoelectronic characteristics of the LiNbO_3_ nanostructure nano-films was determined via using the Ag-decorated LiNbO_3_/Si heterojunction device at the optimum condition. Each of the two devices shows a normal diode operation in both forward and reverse biasing within a rectifying mode. However, the silver-decorated based photodetector showed enhanced forward characteristics. The results were compared with those corresponding to the undecrated LiNbO_3_/Si material. The measurements of I–V in the forward and reverse bias were carried under the dark condition and after doping by heterojunction devices shown in Fig. 12. The two devices depicted the rectifying properties. It is evident that there are two reverse current zones on the I–V curve. The first is the generation zone, where the reverse current marginally rises with the applied voltage and produces electron–hole pairs at low bias. As the reverse bias increases in the second zone, a large rise may be seen. The reverse current of the Ag-decorated LiNbO_3_/Si heterojunction was found to be increased slightly after decoration due to decreasing the resistivity of LiNbO_3_. Two regions are recognized in the I–V characteristics in the forward bias case. The first region represents the recombination current under low voltage, as it is produced when the number of carriers created exceeds the number of self-charging carriers, which occurs when the valence electrons and holes recombine in the conduction band. The mass action law of (nxp > ni^2^) is not valid. The second region, which clearly shows an increase in forward current, indicates the propagation region or the bending under high voltages. For LiNbO_3_/Si heterojunction decorated with Ag, the current could be seen to increase, which is related to decreasing the resistivity of LiNbO_3_ nanostructure compared to the undecorated heterojunction. In fact, the generated dark current comes from the leakage current than passing on the surface of the heterojunction^91^. The ideality factor was found to be around (3.1 and 2.6) for the LiNbO_3_/Si and Ag-decorated LiNbO_3_/Si heterojunction device, respectively. The ideality factors were both higher than the ideal value (1) indicating for non-ideality operation of the pn-heterojunction based photodetector. This can be attributed to the mechanism of tunneling, LiNbO_3_ crystal defects including oxygen vacancies, mismatching between the lattice of LiNbO_3_ and the silicon based wafer, and disorder in surface morphologies^52^. The lower value belonged to the Ag decorated LiNbO_3_ can be as a result of the enhanced built-in potential and barrier height (as the barrier height is inversely proportional to the ideality factor). In addition, as the ideality factor for (Ag decorated LiNbO_3_) was lower than (2), then the transporting process was controlled by the thermionic emission. These values are better than the reported ideality factors in Ref. ^52^. The large ideality factor value confirms the presence of the surface states at the LiNbO_3_/Si interface. Decreasing the ideality factor after doping can be explained due to the compensation effect achieved by Ag decoration which decreases the role of the surface trap.

**Figure 13:**
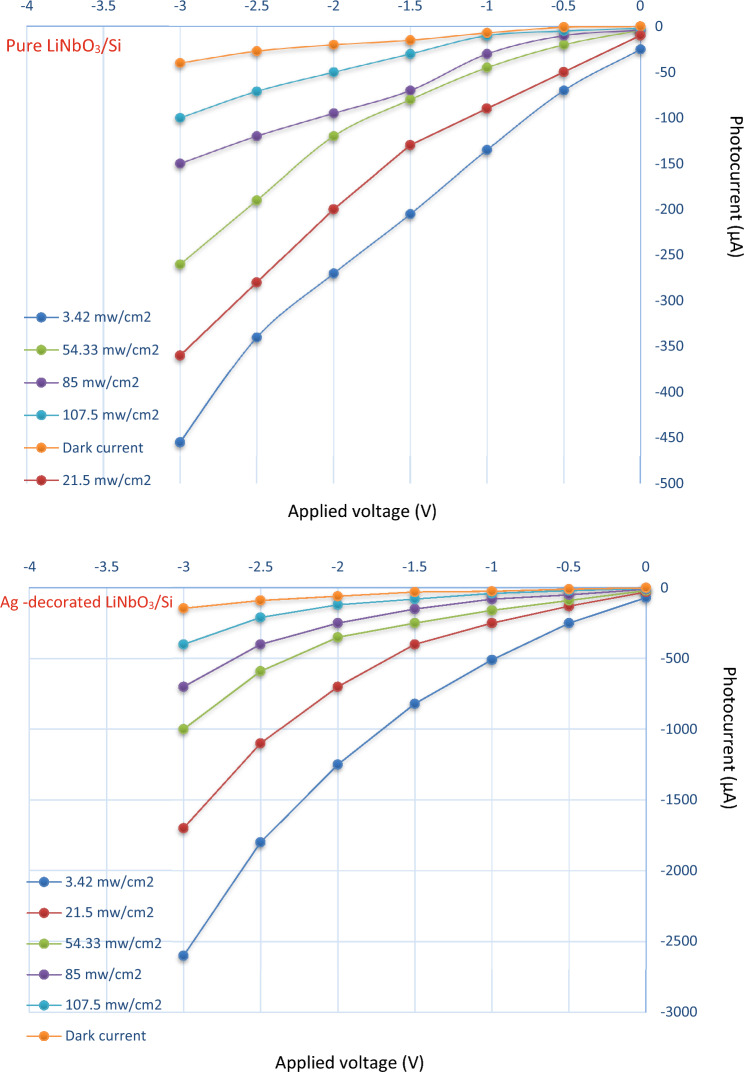
Photocurrent of Pure and Ag-decorated LiNbO_3_/Si heterojunction devices under different levels of illuminated light intensity.

The illuminated I–V characteristics at different illumination intensities are revealed in Fig. 13. The generation of the pairs of electrons-halls in the zone of the depletion and diffusion length is responsible for the rise in photo-current in the reverse bias direction. This occurred when the energy of incident light is greater or equal to the energy gap of LiNbO_3_. The Ag-decorated LiNbO_3_/Si photodetector's structure depicts a clear improvement in the photocurrent. This improvement is connected to the optical absorption's plasmonic impact, which increased the absorption of photons as well as due to widening the depletion layer width owing to the doping effect.

### Capacitance–voltage characteristics

**Figure 14:**
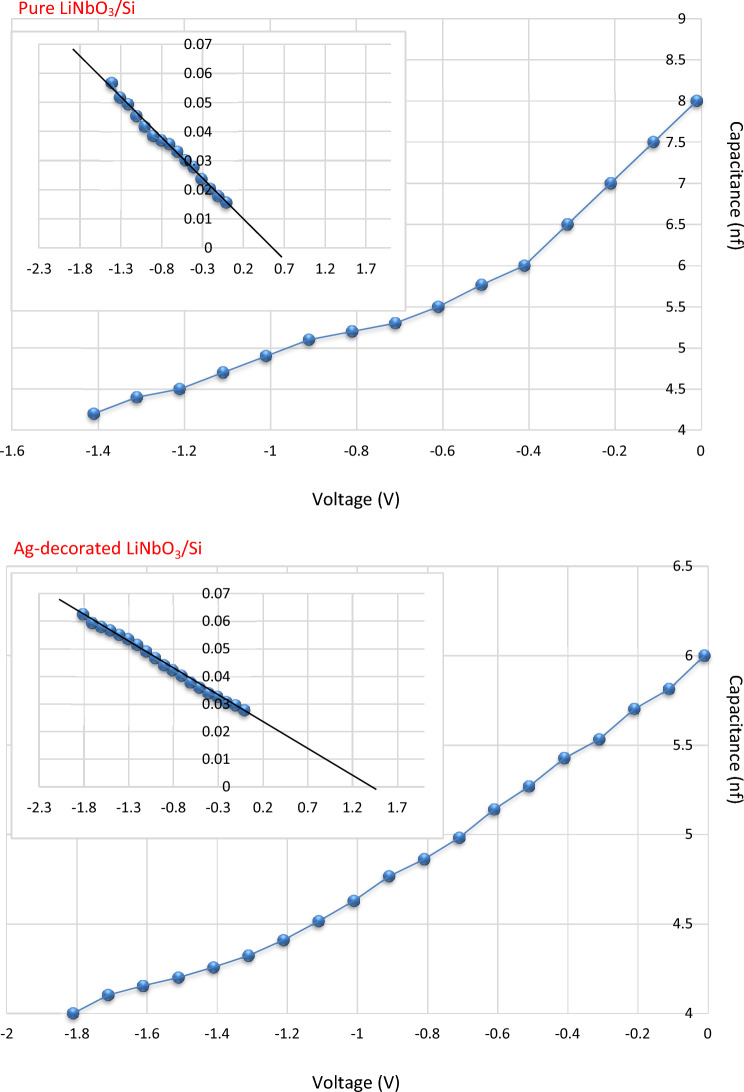
The relation of capacitance–voltage as function for reverse bias characteristics for pure LiNbO_3_/Si and Ag-decorated LiNbO_3_/Si heterojunction.

These properties are essential to the definition of parameters, such as the built-in potential, and the type of the junction. The capacitance (C) is inversely dependent on the reverse bias (Fig. 14). It is clear from this figure that the junction capacitance decreases with the increase of the bias voltage due to increasing of the width of the depletion region. The junction capacitance decreases after the Ag decoration due to the widening of the depletion layer width of the heterojunction. The insert charts of the built-in potential (V_bi_) value of the LiNbO_3_/Si and Ag-decorated LiNbO_3_/Si heterojunction is displayed also in this figure. The value of the built-in potential (V_bi_) was calculated by extrapolating and interception of the straight part of the curve to the zero point of 1/C^2^. The value of Vbi was found to be 0.6 for LiNbO_3_/Si and 1.35 for Ag-decorated LiNbO_3_/Si which agree with^92^. As observed from this figure, the Vbi value increased after the Ag decoration from 0.6 to 1.35 confirming the improvement in the junction properties.

### Spectral responsivity (R_λ_) measurement

**Figure 15:**
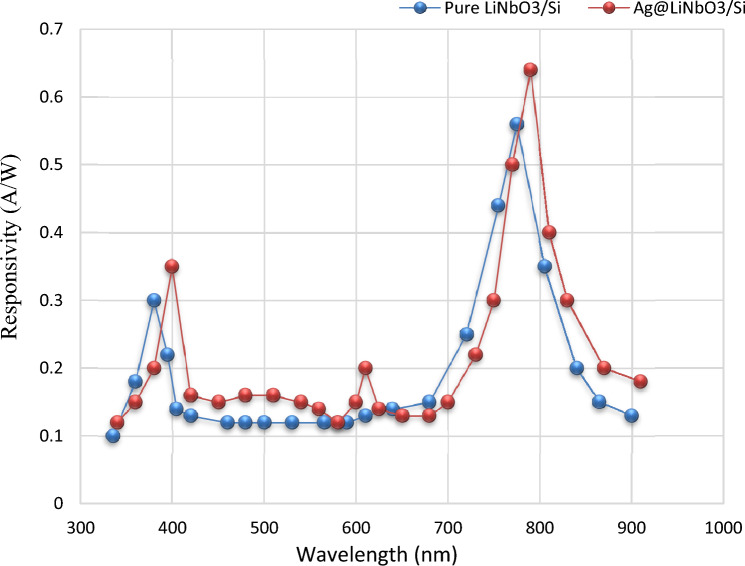
Spectral responsivity of (**a**) pure LiNbO_3_/Si and (**b**) Ag-decorated LiNbO_3_/Si heterojunction device.

The plot of spectral responsivity (R_λ_) as a function of wavelength for LiNbO_3_/Si and Ag-decorated LiNbO_3_/Si heterojunction photo-detector is manifeted in Fig. 15. The increase in responsivity after the decoration with Ag is correlated to the rise in photocurrent arising from increasing the depletion layer width. A slight shifting in the peak response (red shift) towards the long wavelength region could be noticed after the Ag decoration due to the surface Plasmon response (SPR) of Ag. Due to the high absorption coefficient and the band gap of the LiNbO_3_ film, there is a noticeable improvement in responsiveness in the shorter wavelength area. The first small peak was observed at 375 nm due to the absorption edge of the LN films. It may be possible to detect a shift in the peak response at 400 nm, which is connected to a shift in the energy gap for a certain Ag concentration in the direction of the visible spectrum. The second peak was discovered at 790 and related to an increase in the active layer's absorption coefficient brought on by the plasmonic characteristics of the inserted metal. After 900 nm, the low responsivities can be observed as a result of the reduced carriers’ concentrations probabilities. The responsivity of the LiNbO_3_/Si device decorated with Ag was also improved, which may be related to the light absorbed in the depletion region of the silicon substrate^93–95^. Since the minority carrier’s diffusion length is away from the junction interface (depletion zone), these wavelengths are absorbed. In this instance, the incident photon energy's product e–h pairs shift in accordance with the internal electrical field. As a result, the likelihood of carrier concentration might be decreased, which results in lesser responsiveness. On the other hand, the SPR of Ag enhanced the responsivity in visible region around 600 nm.

### Specific detectivity (Dλ*)

**Figure 16:**
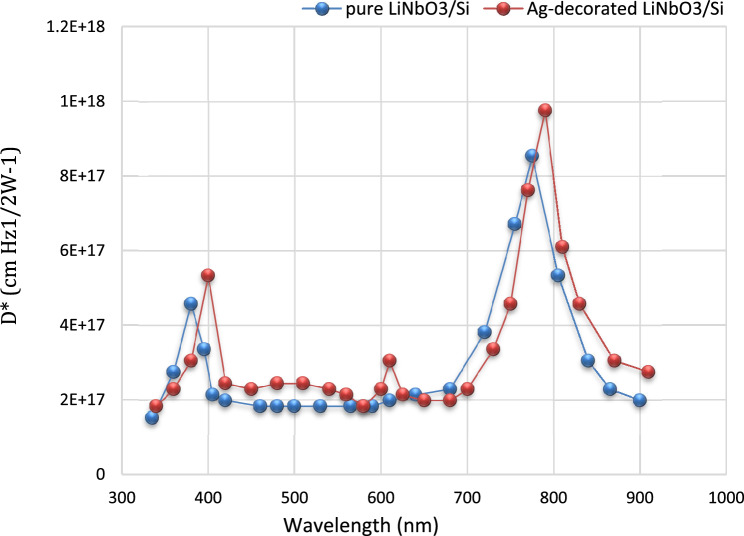
detectivity of pure LiNbO_3_/Si and Ag-decorated LiNbO_3_/Si heterojunction device.

Specific detectivity is a very significant parameter which describes the performance of the photo-detector and its ability to detect the weak signal^96^. The detectivity for this device was calculated by using equation^97–101^:

6 $${\text{D}}* = {\text{ R}}\lambda \, \left( {{\text{A}}.\Delta {\text{f}}} \right)^{{{1}/{2}}} /{\text{I}}_{{\text{n}}} ,$$

7 $${\text{In}} = \, \left( {{\text{2q I}}_{{\text{d}}} .\Delta {\text{f}}} \right)^{{{1}/{2}}} ,$$

where, (Δf): is the frequency band width, (I_d_) is the dark current, and (In) is the noise current. The specific detectivity of the photo-detector depends on the noise current and responsivity. Figure 16 portrays the plot detectivity versus the wavelength for LiNbO_3_/Si and Ag-decorated LiNbO_3_/Si photo-detectors. These figures demonstrate that the detectivity plot is similar to that of responsivity plot. The obtained values of detectivity for the LiNbO_3_/Si and Ag-decorated LiNbO_3_/Si photo-detectors are in the UV visible region. The high detectivity of the device indicates that the photo-detector has low noise, dark current or high responsivity. Enhancement of the detectivity of photo-detector may be attributed to increasing the detector responsivity, decreasing the concentration of structural defects, and decreasing the leakage current^102^.

#### Quantum efficiency (QE)

**Figure 17:**
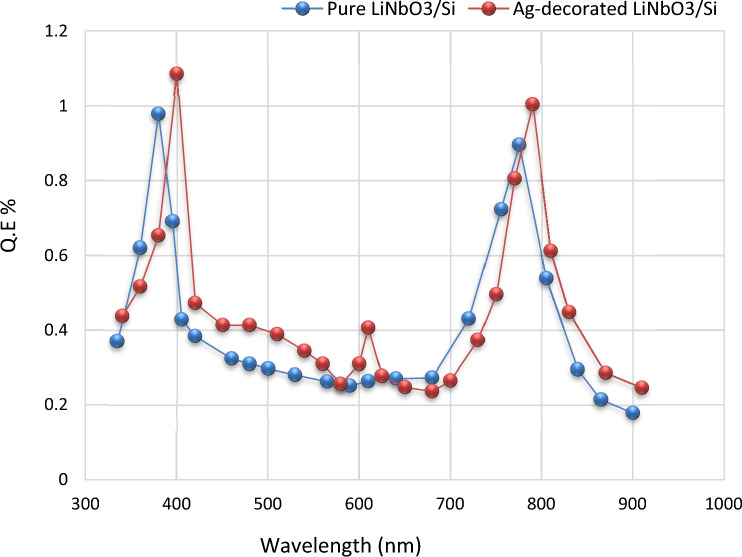
the quantum efficiencies for pure LiNbO_3_/Si and Ag-decorated LiNbO_3_/Si heterojunction devices.

Quantum efficiency can be defined as the ratio between the numbers of generating electron to the incident photon^103^. Figure 17 evinces the quantum efficiency versus the wavelength spectral for the pure LiNbO_3_/Si and Ag decorated LiNbO_3_/Si photo-detectors. Quantum efficiency was estimated by the equation^104–107^:

8 $${\text{Q }} = { 1}.{\text{24 R}}_{\lambda } /\lambda \, \left( {\mu {\text{m}}} \right).$$

The highest value was at a (390 nm) wavelength for Ag decorated LiNbO_3_/Si and at a (375 nm) wavelength for LiNbO_3_/Si photo-detector. The value of obtained quantum efficiency is larger or comparable to the wide band gap silicon-based heterojunction^98–111^. The enhancement was due to the incorporation of silver (Ag) noble metallic nanoparticles that induced a plasmonic effect leading to higher extraction of electrons generated via incident photon.

## Conclusion

At various doping periods, the effects of incorporating silver nanoparticles (Ag) on the structural, optical, morphological, and electrical characteristics of LiNbO_3_ nanostructure were investigated. In comparison to the pure LiNbO_3_ film, the SEM pictures showed a dense distribution of grains with novel structure at a higher time of 45 s. In this study, ornamented Ag significantly boosted and improved the device photoresponse and photocurrent.

## Supplementary Information

Supplementary Information.

## Data Availability

Correspondence and requests for materials should be addressed to Evan T. Salim, Rawan B. Fadhil, Makram A. Fakhri.
