# Supplementary material for: Silver decorated lithium niobat nanostructure by UV activation method for silver–lithium niobate/silicon heterojunction device
Source: Sci Rep. 2023 Jul 17;13:11514. doi: 10.1038/s41598-023-38363-8 (PMC10352337; doi:10.1038/s41598-023-38363-8)
Supplement: Supplementary file 1 — Supplementary Information. [file 41598_2023_38363_MOESM1_ESM.pdf]

SPRINGER NATURE

Change of authorship request form - Journals (pre-acceptance)

Important information. Please read.

- This form should be used by authors to request any change in authorship (adding/deleting authors) including changes in corresponding authors. This form should not be used for name changes. Please fully complete all sections. Use black ink and block capitals and provide each author's full name with the given name first followed by the family name.

- By signing this declaration, all authors guarantee that the order of the authors are in accordance with their scientific contribution, if applicable as different conventions apply per discipline, and that only authors have been added who made a meaningful contribution to the work.

- Please note, in author collaborations where there is formal agreement for representing the collaboration, it is sufficient for the representative or legal guarantor (usually the corresponding author) to complete and sign the Authorship Change Form on behalf of all authors, **next to the added/removed author(s)**. **(Complete Section 3, followed by Section 6.)**
  In author collaborations where there is no formal agreement for representing the collaboration and **there are more than 10 authors**, one may sign for all, provided the signer appends correspondence that attests that each of the authors have agreed to the change **and the added/removed authors sign the form**. **(Complete Section 3, followed by Section 6.)**

- Please note, we cannot investigate or mediate any authorship disputes. If you are unable to obtain agreement from all authors (including those who you wish to be removed) you must refer the matter to your institution(s) for investigation. Please inform us if you need to do this.

- If you are not able to return a fully completed form within **30 days** of the date that it was sent to the author requesting the change, we may have to withdraw your manuscript. We cannot publish manuscripts where authorship has not been agreed by all authors (including those who have been removed).

- Incomplete forms will be rejected.

- Please return/upload this form, fully completed, to the Journals Editorial Office. The Journal and/or Publisher will consider the information you have provided to decide whether to approve the proposed change in authorship. We may decide to contact your institution for more information or undertake a further investigation, if appropriate, before making a final decision.

SPRINGER NATURE

Change of authorship request form - Journals (pre-acceptance)

Section 1: Please provide the current title of manuscript

Manuscript ID no.: Submission ID b6a16f1d-98fb-457a-bfde-98c678481020

Title: **Ag-decorated lithium niobate nanostructure by UV activation method for Ag– LiNbO 3 /Si heterojunction device**

Section 2: Please provide the previous authorship, in the order shown on the manuscript before the changes were introduced. Please indicate the corresponding author by adding (CA) behind the name.

	First name(s)	Family name	ORCID or SCOPUS id, if available
1st author	**Evan**	**Salim**	**ORCID: 0000-0001-6983-5952**
2nd author	**Rawan**	**Fadhil**	
3rd author	**Makram**	**Fakhri**	**ORCID:0000-0001-9010-6121**
4th author	**Wafaa**	**Khalef**	
5th author	**Ahmad**	**Alzzahrani**	
6th author	**Raed**	**Ibrahim**	
7th author	**Raid**	**Ismail**	
8th author			
9th author			
10th author			

Please use an additional sheet if there are more than 10 authors.

SPRINGER NATURE Springer Nature is one of the world's leading global research, educational and professional publishers, created in May 2015 through the combination of Nature Publishing Group, Palgrave Macmillan, Macmillan Education and Springer Science+Business Media.

SPRINGER NATURE

Change of authorship request form - Journals (pre-acceptance)

Section 3: Please provide a justification for change. Please use this section to explain your reasons for changing the authorship of your manuscript, e.g. what necessitated the change in authorship? Please refer to the (journal) policy pages for more information about authorship. Please explain why omitted authors were not originally included and/or why authors were removed on the submitted manuscript.

Dear Sir: We had to change the names because of our University instructions, as Prof. Wafaa is an assistant supervisor, and must here name in the first three authors.

Section 4: Proposed new authorship. Please provide your new authorship list in the order you would like it to appear on the manuscript. Please indicate the corresponding author by adding (CA) behind the name. If the Corresponding Author has changed, please indicate the reason under section 3.

	First name(s)	Family name (this name will appear in full on the final publication and will be searchable in various abstract and indexing databases)	Affiliated institute	E-mail address
1st author	Evan	Salim	University of Tecvhnology-Iraq	evan_tarq@yahoo.com
2nd author	Wafaa	Khalaf	University of Technology-Iraq	drwafaa1980@gmail.com
3rd author	Makram	Fakhri	University of Technology-Iraq	mokaram_76@yahoo.com
4th author	Rawan	Fadhil	University of Technology-Iraq	rawanbashaar.97@gmail.com
5th author	Ahmad	Azzahrani	Northern Border University, Arar, KSA	ahmad.azzahrani@nbu.edu.sa
6th author	Raed	Ibrahim	AlFarahidi University	raed.khalid@alfarahidiuc.edu.iq
7th author	Raid	Ismail	University of Technology-Iraq	raidismail@yahoo.com
8th author				
9th author				
10th author				

Please use an additional sheet if there are more than 10 authors.

SPRINGER NATURE Springer Nature is one of the world's leading global research, educational and professional publishers, created in May 2015 through the combination of Nature Publishing Group, Palgrave Macmillan, Macmillan Education and Springer Science+Business Media.

SPRINGER NATURE

Change of authorship request form - Journals (pre-acceptance)

Section 5: Author contribution, Acknowledgement and Disclosures. Please use this section to provide a new disclosure statement and, if appropriate, acknowledge any contributors who have been removed as authors and ensure you state what contribution any new authors made (if applicable per the journal or book (series) policy). Please ensure these are updated in your manuscript - after approval of the change(s) - as our production department will not transfer the information in this form to your manuscript.

New acknowledgements:

- **The authors would like to thank the Appliedc Science department-University of Technology for the logistic support this work.**
- **The authors gratefully thank the Prince Faisal bin Khalid bin Sultan Research Chair in Renewable Energy Studies and Applications (PFCRE) at Northern Border University for their support and assistance.**

New Disclosures (financial and non-financial interests, funding):

The authors extend their appreciation to the Deanship of Scientific Research at Northern Border University, Arar, KSA for funding this research work through the project number "NBU-FFR-2023-0057

New Author Contributions statement (if applicable per the journal policy):

Author Contributions
Conceptualization, Evan T. Salim, Wafaa K. Khalef; Makram A. Fakhri, Rawan B. Fadhil, methodology, Evan T. Salim, Rawan B. Fadhil, Makram A. Fakhri; validation, Evan T. Salim, Wafaa K. Khalef; Makram A. Fakhri, Rawan B. Fadhil, Ahmad S. Azzahrani, Raed Khalid Ibrahim, Raid A. Ismail; formal analysis, Evan T. Salim, Wafaa K. Khalef , Makram A. Fakhri, Rawan B. Fadhil; investigation, Evan T. Salim, Wafaa K. Khalef, Makram A. Fakhri, Rawan B. Fadhil, Ahmad S. Azzahrani, Raed Khalid Ibrahim, Raid A. Ismail; resources, Evan T. Salim, Wafaa K. Khalef , Makram A. Fakhri, Ahmad S. Azzahrani, Raed Khalid Ibrahim, Raid A. Ismail; data curation, Evan T. Salim, Wafaa K. Khalef, Makram A. Fakhri, Rawan B. Fadhil; writing—original draft preparation, Rawan B. Fadhil; writing—review and editing, Evan T. Salim, Wafaa K. Khalef, Makram A. Fakhri, Ahmad S. Azzahrani, Raed Khalid Ibrahim, Raid A. Ismail; visualization, Evan T. Salim, Makram A. Fakhri, Ahmad S. Azzahrani, Raed Khalid Ibrahim, Raid A. Ismail; supervision, Evan T. Salim, Wafaa K. Khalef; project administration, Evan T. Salim, Wafaa K. Khalef, Makram A. Fakhri; funding acquisition, Ahmad S. Azzahrani, All authors have read and agreed to the published version of the manuscript.

State 'Not applicable' if there are no new authors.

SPRINGER NATURE Springer Nature is one of the world's leading global research, educational and professional publishers, created in May 2015 through the combination of Nature Publishing Group, Palgrave Macmillan, Macmillan Education and Springer Science+Business Media.

SPRINGER NATURE

Change of authorship request form - Journals (pre-acceptance)

Section 6: Declaration of agreement. *All* authors, unchanged, new and removed *must* sign this declaration.
(NB: Please print the form, (docu)-sign and return/upload a scanned copy. Please note that signatures that have been inserted as an image file are acceptable as long as it is handwritten. Typed names in the signature box are unacceptable.) *** Please delete as appropriate. Delete all of the bold if you were on the original authorship list and are remaining as an author.**

	First name	Family name		Signature	Date
1st author	**Evan**	**Salim**	I agree to the proposed new authorship shown in section 4 **/and the addition/removal*of my name to the authorship list** /and the proposed change in corresponding author		**11-06-2021**
2nd author	**Wafaa**	**Khalef**	I agree to the proposed new authorship shown in section 4 **/and the addition/removal*of my name to the authorship list** /and the proposed change in corresponding author		**13-06-2023**
3rd author	**Makram**	**Fakhri**	I agree to the proposed new authorship shown in section 4 **/and the addition/removal*of my name to the authorship list** /and the proposed change in corresponding author		**11-06-2023**
4th authors	**Rawan**	**Fadhil**	I agree to the proposed new authorship shown in section 4 **/and the addition/removal*of my name to the authorship list** /and the proposed change in corresponding author		**12-06-2023**
5th author	**Ahmad**	**Alzzahrani**	I agree to the proposed new authorship shown in section 4 **/and the addition/removal*of my name to the authorship list** /and the proposed change in corresponding author		**11-06-2023**
6th author	**Raed**	**Ibrahim**	I agree to the proposed new authorship shown in section 4 **/and the addition/removal*of my name to the authorship list** /and the proposed change in corresponding author		**12-06-2023**
7th author	**Raid**	**Ismail**	I agree to the proposed new authorship shown in section 4 **/and the addition/removal*of my name to the authorship list** /and the proposed change in corresponding author		**12-06-2023**

SPRINGER NATURE Springer Nature is one of the world's leading global research, educational and professional publishers, created in May 2015 through the combination of Nature Publishing Group, Palgrave Macmillan, Macmillan Education and Springer Science+Business Media.

	First name	Family name		Signature	Date
8th author			I agree to the proposed new authorship shown in section 4 /**and the addition/removal*of my name to the authorship list** /and the proposed change in corresponding author		
9th author			I agree to the proposed new authorship shown in section 4 /**and the addition/removal*of my name to the authorship list** /and the proposed change in corresponding author		
10th author			I agree to the proposed new authorship shown in section 4 /**and the addition/removal*of my name to the authorship list** /and the proposed change in corresponding author		

Please use an additional sheet if there are more than 10 authors.

In case of author collaborations with formal agreement:

	Name of consortium/consortia	First name	Family name		Signature	Date
Representative/legal guarantor				I agree to the proposed new authorship shown in section 4 /**and the addition/removal*of my name to the authorship list** /and the proposed change in corresponding author		

Both added/removed authors should complete the information in the first table under Section 6.

---- End of form ----

SPRINGER NATURE Springer Nature is one of the world’s leading global research, educational and professional publishers, created in May 2015 through the combination of Nature Publishing Group, Palgrave Macmillan, Macmillan Education and Springer Science+Business Media.
